# The Long-Term Effect of Preterm Birth on Renal Function: A Meta-Analysis

**DOI:** 10.3390/ijerph18062951

**Published:** 2021-03-13

**Authors:** Ju Sun Heo, Jiwon M. Lee

**Affiliations:** 1 Department of Pediatrics, Anam Hospital, Korea University College of Medicine, Seoul 02841, Korea; heojs08@korea.ac.kr; 2 Department of Pediatrics, Chungnam National University Hospital, Daejeon 35015, Korea; 3 Department of Pediatrics, Chungnam National University College of Medicine, Daejeon 35015, Korea

**Keywords:** preterm, long-term, renal function, prematurity, meta-analysis

## Abstract

The preterm-born adult population is ever increasing following improved survival rates of premature births. We conducted a meta-analysis to investigate long-term effects of preterm birth on renal function in preterm-born survivors. We searched PubMed and EMBASE to identify studies that compared renal function in preterm-born survivors and full-term-born controls, published until 2 February 2019. A random effects model with standardized mean difference (SMD) was used for meta-analyses. Heterogeneity of the studies was evaluated using Higgin’s *I*^2^ statistics. Risk of bias was assessed using the Newcastle–Ottawa quality assessment scale. Of a total of 24,388 articles screened, 27 articles were finally included. Compared to full-term-born controls, glomerular filtration rate and effective renal plasma flow were significantly decreased in preterm survivors (SMD −0.54, 95% confidence interval (CI), −0.85 to −0.22, *p* = 0.0008; SMD −0.39, 95% CI, −0.74 to −0.04, *p* = 0.03, respectively). Length and volume of the kidneys were significantly decreased in the preterm group compared to the full-term controls (SMD −0.73, 95% CI, −1.04 to −0.41, *p* < 0.001; SMD −0.82, 95% CI, −1.05 to −0.60, *p* < 0.001, respectively). However, serum levels of blood urea nitrogen, creatinine, and cystatin C showed no significant difference. The urine microalbumin to creatinine ratio was significantly increased in the preterm group. Both systolic and diastolic blood pressures were also significantly elevated in the preterm group, although the plasma renin level did not differ. This meta-analysis demonstrates that preterm-born survivors may be subject to decreased glomerular filtration, increased albuminuria, decreased kidney size and volume, and hypertension even though their laboratory results may not yet deteriorate.

## 1. Introduction

The incidence rate of preterm births was about 11.1% of all livebirths worldwide, and the burden of preterm births has increased in recent decades; about 13 million infants are born preterm each year [1,2]. With recent improvement in perinatal and neonatal care, the survival rates in preterm babies have increased [3]. According to a recent report, more than 95% of preterm-born patients survive into adulthood [4]. In addition, as the first generation of extremely preterm infants reaches young adulthood and their numbers increase, there is an increasing interest to investigate the long-term prognosis of organ function in the preterm-born population, including renal function [5,6].

Nephrogenesis in humans starts from the 20th week of gestation and more than half of the total number of nephrons develop in the last three months of pregnancy, up until 36 weeks [7]. Prematurity, defined as a birth before 37 weeks of gestation, may occur at critical stages of late nephronal development. Although postnatal nephrogenesis continues up to 40 days after preterm birth, the postnatal development can be altered or sometimes abnormal [8,9,10]. Moreover, Sutherland and colleagues studied autopsied kidneys from preterm neonates and demonstrated that preterm kidneys had a decreased number of functional nephrons [11]. It has also been reported that the number of nephrons is proportional to the gestational age (GA) [9], and a decrease in the number of nephrons could be subject to increased risk of developing hypertension and chronic kidney disease (CKD) in later life [12]. Therefore, long-term follow-up of kidney function is essential for preterm infants.

There have been several studies demonstrating the relationship between low birth weight and CKD [13,14,15]. Low birth weight has been known as a risk group for CKD in childhood and adulthood. However, almost all studies did not investigate gestational age. Relatively few studies have examined the effects of preterm birth on the subsequent risk of CKD. A recent large-scaled national cohort study demonstrated that preterm birth is a strong risk factor for the development of CKD from childhood into mid-adulthood [16]. However, there have been no more detailed clinical data to validate CKD diagnosis. For clinicians who should monitor kidney function for a long time in preterm-born patients, the important issue is the changes in more detailed clinical data for kidney function in preterm-born patients.

Here, we conducted a meta-analysis of current studies published to date, in order to investigate the long-term effect of preterm birth on more detailed renal function data including laboratory biomarkers, sonographic data, and blood pressure.

## 2. Materials and Methods

### 2.1. Literature Search and Study Selection

**Figure 1:**
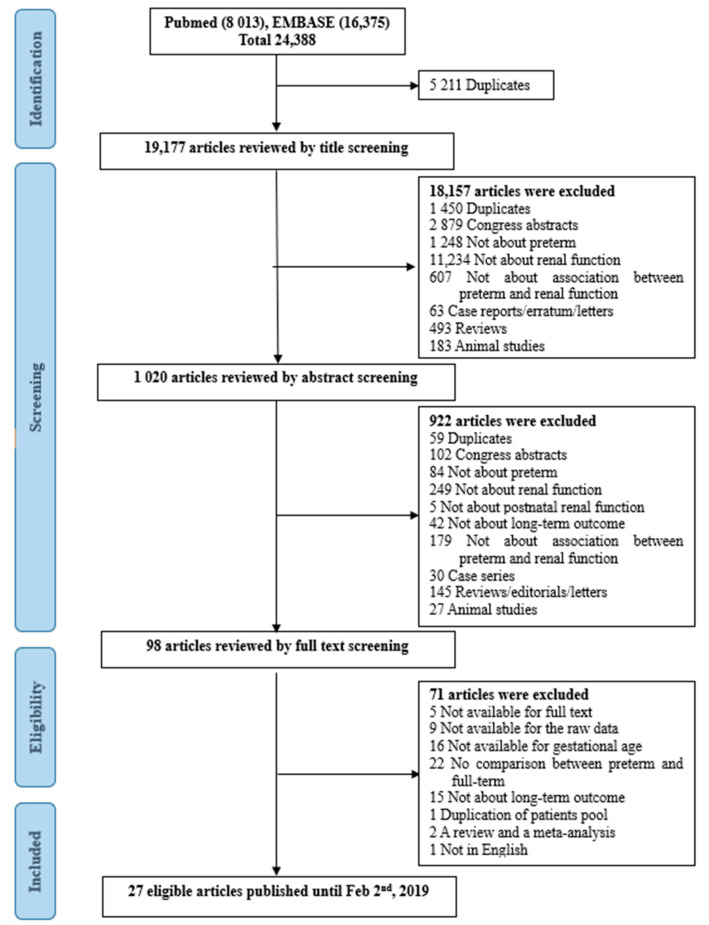
Flow chart of the literature search.

We conducted PubMed and EMBASE searches to identify eligible articles (Supplementary Table S1). Literature published until 2 February 2019 was searched. The search terms included: (infant, preterm OR infant, premature OR low birth weight) AND (kidney function* OR kidney failure* OR kidney disease* OR kidney insufficien* OR renal function* OR renal failure* OR renal disease* OR renal insufficien* OR glomerular filtration rate* OR hypertension OR proteinuria OR microalbuminuria OR nephron*). The language was limited to English. The complete search strategy is shown in Table S1. Records were managed by the EndNote X8.0 software (Clarivate Analytics, Philadelphia, PA, USA) to remove duplicates. Publications were screened first by title, second by abstract, and finally by full text, based on our eligibility criteria (Figure 1).

### 2.2. Inclusion and Exclusion Criteria

We included cohort, case–control, or cross-sectional studies which compared long-term kidney function between preterm and full-term controls. Preterm infants were defined as the GA below 37 weeks including small for GA (SGA, i.e., birth weight <10th percentile for GA) and appropriate for GA (AGA, i.e., birth weight 10th–90th percentile for GA). We only included studies with results of kidney function evaluations conducted above postnatal age 24 months. The exclusion criteria were as follows: (1) studies that included low-birth weight infants without the mention of GA; (2) animal studies, case series, review articles, and articles without applicable data; (3) studies that included specific diseases such as congenital anomalies, IgA nephropathy, minimal change nephropathy, focal segmental glomerulosclerosis, and diabetic nephropathy.

### 2.3. Data Extraction and Outcomes

Two reviewers (J.S.H., and J.M.L.) extracted eligible studies independently through the review of titles, abstracts, and full texts. In case of disagreement, a final decision was made by consensus. Data extraction was carried out as recommended by the Cochrane handbook [17] and included authors, year of publication, participants, demographic characteristics, age at follow-up study, renal function-related markers (serum creatinine (SCr), blood urea nitrogen (BUN), cystatin C, glomerular filtration rate (GFR), renin, effective renal plasma flow (ERPF), urine albumin to creatinine ratio (uACR)), kidney length, kidney volume, relative kidney volume, and blood pressure. GFR using the Schwartz formula was calculated as (*k* × Height (cm))/SCr (mg/dL), where *k* = 0.45 for infants <1 year, 0.55 for children from 2 to 13 years and adolescent girls, and 0.70 for adolescent boys. ERPF was measured as the clearance of para-aminohippuric acid. Sonographic measurements were performed with the subject lying in the supine position and scanned in the para-coronal view with the transducer positioned to obtain the longest kidney dimension. Kidney volume was calculated using the formula: (kidney length × kidney width × kidney thickness) × π/6. Relative kidney volume was calculated by dividing renal volume by body surface area. The lengths and volume of the kidneys were calculated as the average of both kidneys. The data of blood pressure included ambulatory blood pressure monitoring (ABPM) as well as systolic and diastolic blood pressures (SBP and DBP).

Kidney function data were collected as mean ± standard deviation (SD). Where the data were given in median and interquartile ranges (IQR), we used the quantile method for estimating the mean and SD from the median and IQR, proposed by Wan and colleagues [18].

(1) $$Mean\approx \frac{q1+m+q3}{3}\;SD\approx \frac{q3-q1}{1.35}$$

where *q*1 = first quartile, *m* = median, *q*3 = third quartile.

### 2.4. Quality Assessment

This meta-analysis was conducted and reported according to the Preferred Reporting Items for Systematic Reviews and Meta-analyses (PRISMA) individual patient data (IPD) guidelines [19]. For assessment of risk of bias of individual studies, the Newcastle–Ottawa scale (NOS) for non-randomized studies was used [20]. The scoring was performed independently by two reviewers (J.S.H., J.M.L.). We used a 9-point system to evaluate the NOS scores. A study score of 7–9 or above was considered high quality, a score of 4–6 was considered medium quality, and a score of 0–4 or below was considered low quality.

### 2.5. Statistical Analysis and Evaluation of Heterogeneity and Publication Bias

In the meta-analysis, the standardized mean difference (SMD) method and corresponding 95% confidence intervals (CIs) were used to compare the kidney function data between preterm and full-term infants. If the preterm group was divided into SGA and AGA subgroups, we combined two subgroups into a single preterm group by using the formulae suggested by the Cochrane handbook for systematic reviews of interventions [17].

Sample size = N_1_ + N_2_ (2)

(3) $$\mathrm{Mean}=\frac{N_{1}M_{1}+N_{2}M_{2}}{N_{1}+N_{2}}$$

(4) $$\mathrm{SD}=\;\sqrt{\frac{\left(N_{1}-1\right){\mathrm{SD}}_{1}^{2}+\left(N_{2}-1\right){\mathrm{SD}}_{2}^{2}+\frac{N_{1}N_{2}}{N_{1}+N_{2}}\left(M_{1}^{2}+M_{2}^{2}-2M_{1}M_{2}\right)}{N_{1}+N_{2}-1}}\;(N_{1}\;=\;\mathrm{sample}\;\mathrm{size}\;\mathrm{of}\;\mathrm{SGA}\;\mathrm{group},\;N_{2}\;=\;\mathrm{sample}\;\mathrm{size}\;\mathrm{of}\;\mathrm{AGA}\;\mathrm{group},\;M_{1}\;=\;\mathrm{mean}\;\mathrm{of}\;\mathrm{SGA}\;\;\mathrm{group},\;M_{2}\;=\;\mathrm{mean}\;\mathrm{of}\;\mathrm{AGA}\;\mathrm{group},\;{\mathrm{SD}}_{1}\;=\;\mathrm{standard}\;\mathrm{deviation}\;\mathrm{of}\;\mathrm{SGA}\;\mathrm{group},\;{\mathrm{SD}}_{2}\;=\;\;\mathrm{standard}\;\mathrm{deviation}\;\mathrm{of}\;\mathrm{AGA}\;\mathrm{group})$$

Random effects models were used because of the heterogeneity of the included studies. We assessed the heterogeneity of the studies by using the Cochran Q test, and a *p*-value of <0.1 was considered significant [21,22]. The inconsistency across the studies was also measured by the *I*^2^ metric, as a measure of the percentage of total variation across the studies because of the heterogeneity [23]. *I*^2^ values of <25, 25–75, and >75% were considered to represent low, moderate, and high levels of heterogeneity, respectively. Studies with high disparity were removed from analysis to control high heterogeneity.

Publication bias of each article was estimated by inspecting the funnel plot and using the Egger test when there were 10 or more eligible studies. All analyses were conducted using RevMan 5.4 (The Nordic Cochrane Centre).

## 3. Results

### 3.1. Study Selection, Qualitiative and Quantitative Analysis

A total of 24,388 articles were identified using electronic and manual research. There were 5211 duplicates. After serially reviewing the titles, abstracts, and full texts, 27 eligible studies were finally selected [8,24,25,26,27,28,29,30,31,32,33,34,35,36,37,38,39,40,41,42,43,44,45,46,47,48,49]. The detailed process of article selection is shown in Figure 1. The 27 articles included 4804 patients with 1699 preterm-born patients and 3105 full-term infants. There were four respective studies from two cohorts, each of which investigated different factors with a few years’ interval from the other studies [38,42,45,47].

Baseline characteristics of selected studies are presented in Table 1. The range of GA and birth weight for preterm infants were 25–35 weeks of gestation and 724–3045 g, respectively. The range of age at follow-up for kidney function was 6.6–49 years. Studies evaluated renal function in various aspects, including biomarkers, such as SCr and cystatin C, metrics using radiologic images, such as kidney lengths and volumes, and blood pressures.

The PRISMA checklist for the meta-analysis is shown in Table S2. The study quality assessed by using the NOS can be seen in Table S3. The overall score was medium–high with an average of 6.5 out of a maximum of 9 points. The study quality scored 5 in 1 study, 6 in 15 studies, 7 in 8 studies, 8 in 2 studies, and 9 in 1 study (range, 1 [very poor] to 9 [very high]). Controls did not come from the same population as the cases in 85.1% of studies. Comparability of groups on the basis of design or analysis for possible confounding factors was absent or not sufficiently stated in 59.2% of studies, and information about non-response rates was insufficient or not stated in 92.5% of studies.

### 3.2. Meta-Analysis of Renal Function-Related Markers in Preterm Infants Compared to Full-Term Controls

In the selected studies, various markers related to renal function were investigated. These included serum markers, such as SCr, BUN, cystatin C, GFR, and renin, a marker related to renal hypertension. In addition, ERPF and uACR were also evaluated. We performed a meta-analysis for these renal function-related markers.

**Figure 2:**
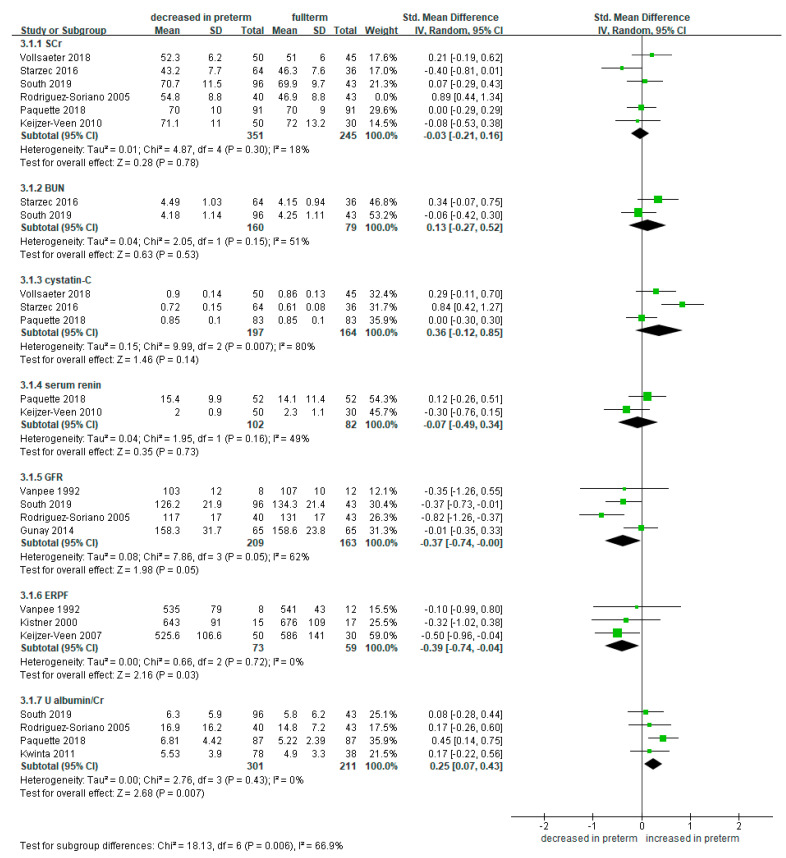
Forest plot of meta-analysis of renal function-related markers in preterm infants compared to full-term controls. Some studies with high disparity were removed from analysis to control high heterogeneity. Abbreviations: BUN, blood urea nitrogen; CI, confidence interval; ERPF, effective renal plasma flow; GFR, glomerular filtration rate; SCr, serum creatinine; SD, standard deviation; Std, standardized; U albumin/Cr, urine albumin to creatinine ratio.

In meta-analyses of SCr (studies = 6) [8,24,25,28,38,44], BUN (studies = 2) [8,28], cystatin C (studies = 3) [24,25,28], and renin (studies = 2) [25,38], there were no significant differences in the level of all these markers between the preterm and full-term infants (Figure 2). In addition, there were four studies which measured and compared GFR [8,34,44,49]. GFR levels were lower in the preterm infants compared to the full-term controls (SMD −0.54, 95% CI −0.85 to −0.22; participants = 372; 209 were preterm infants and 163 were full-term controls; *I*^2^ = 18%). Of the four studies included in the meta-analysis, two reported a significant decrease [8,44], and the other two reported insignificance [34,49]. Three studies reported on the ERPF levels, which were significantly lower in the preterm infants compared to the full-term controls (SMD −0.39, 95% CI −0.74 to −0.04; participants = 132; 73 were preterm infants and 59 were full-term controls; *I*^2^ = 0%) [42,47,49]. Four studies investigated uACR [8,25,36,44]. uACR levels were significantly higher in the preterm infants compared to the full-term controls (SMD 0.25, 95% CI 0.07 to 0.43; participants = 512; 301 were preterm infants and 211 were full-term controls; *I*^2^ = 0%) (Figure 2).

### 3.3. Meta-Analysis of Kidney Length and Volume in Preterm Infants Compared to Full-Term Controls

**Figure 3:**
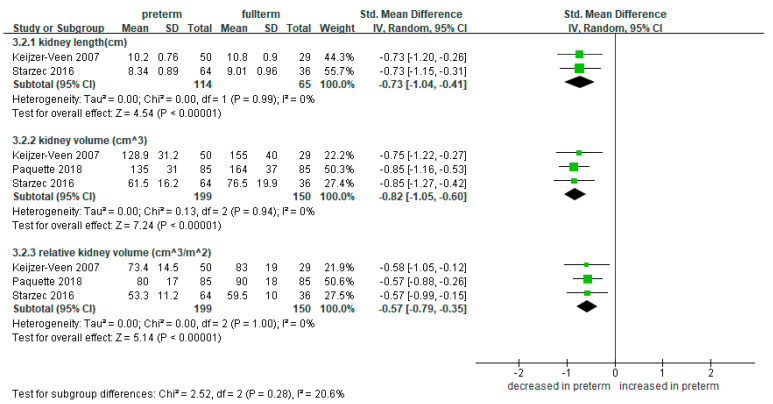
Forest plot of meta-analysis of kidney lengths and volumes compared in preterm and full-term groups. Abbreviations: CI, confidence interval; SD, standard deviation; Std, standardized.

Other than the markers, three studies tried to evaluate the renal mass itself, by measuring the lengths and volumes of the kidneys [25,28,42]. Two studies measured the lengths of the kidneys [28,42], and the meta-analysis showed that preterm infants were significantly shorter in kidney length compared to the full-term controls (SMD −0.73, 95% CI −1.04 to −0.41; 114 preterm-born and 65 full-term controls; *I*^2^ = 0%) (Figure 3). Three studies reported on the absolute and relative kidney volumes calibrated by the body surface area in 199 preterm- and 150 full-term-born patients [25,28,42]. The preterm-born had significantly smaller renal volume compared to the full-term controls in both absolute (SMD −0.82, 95% CI −1.05 to −0.60; 199 preterm-born and 150 full-term controls) and relative (SMD −0.57, 95% CI −0.79 to −0.35; 199 preterm-born and 150 full-term controls) renal volumes (Figure 3).

### 3.4. Meta-Analysis of Blood Pressure in Preterm Infants Compared to Full-Term Controls

We compared the SBP and DBP in preterm- and full-term-born groups. Compared to other items, blood pressure was measured and compared in several studies. A total of 20 studies measured and compared the blood pressure in groups between preterm- and full-term-born [8,24,25,26,27,29,30,31,32,33,37,38,39,40,41,43,44,46,47,48]. Of the 20 studies, 8 studies performed ABPM [25,29,31,32,38,39,46,47].

**Figure 4:**
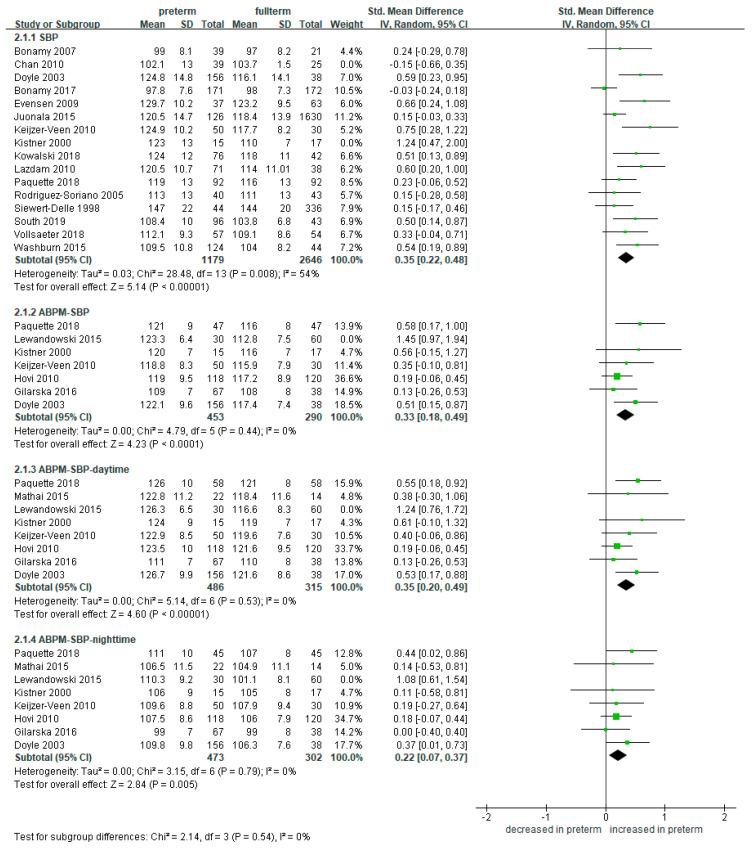
Forest plot of meta-analysis of the systolic blood pressure compared in preterm and full-term groups. Some studies with high disparity were removed from analysis to control high heterogeneity. Abbreviations: ABPM, ambulatory blood pressure monitoring; CI, confidence interval; SBP, systolic blood pressure; SD, standard deviation; Std, standardized.

The SBP was significantly higher in the 1233 preterm-born patients compared to the 2688 full-term-born counterparts (SMD 0.36, 95% CI 0.22 to 0.48) (Figure 4). The trend was consistent for both single-time measured and ambulatory monitored studies. ABPM-SBP was more elevated in the preterm-born group than the full-term group both for the daytime (SMD 0.33, 95% CI 0.18 to 0.49; 516 preterm-born and 375 full-term controls) and the nighttime (SMD 0.35, 95% CI 0.20 to 0.49; 503 preterm-born and 362 full-term controls).

**Figure 5:**
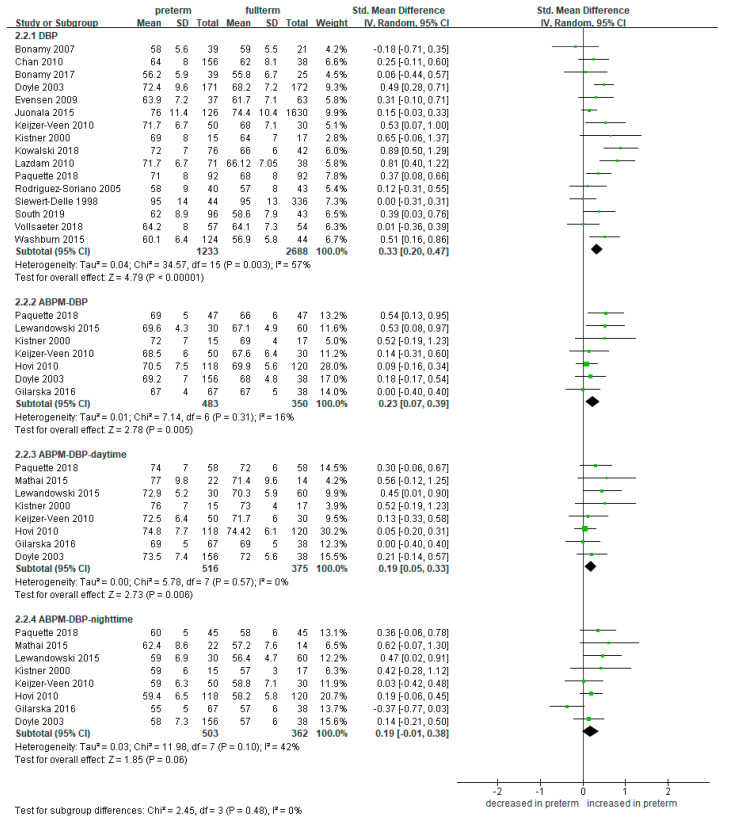
Forest plot of meta-analysis of the diastolic blood pressure compared in preterm and full-term groups. Abbreviations: ABPM, ambulatory blood pressure monitoring; CI, confidence interval; DBP, diastolic blood pressure; SD, standard deviation; Std, standardized.

Moreover, the DBP was also significantly elevated in the 1233 preterm-born compared to the 2688 full-term controls collected from 16 studies (SMD 0.33, 95% CI 0.20 to 0.47) (Figure 5) [8,24,25,26,27,30,33,37,38,40,41,43,44,46,47,48]. ABPM-DBP showed the same trend in the daytime (SMD 0.19, 95% CI 0.05 to 0.33; 516 preterm-born and 375 full-term controls) and in the nighttime (SMD 0.25, 95% CI 0.21 to 0.36; 503 preterm-born and 362 full-term controls).

### 3.5. Subgroup Analysis According to SGA and AGA

Due to the limited number of studies that separate SGA and AGA, subgroup analysis could only be performed for the blood pressure and SCr.

#### 3.5.1. SGA

**Figure 6:**
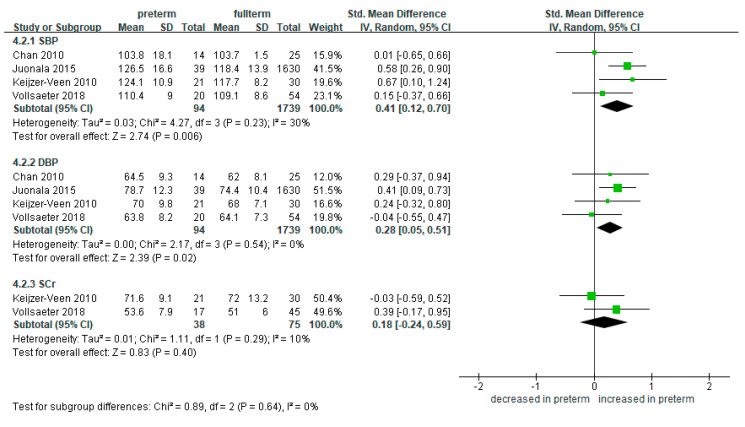
Forest plot of subgroup meta-analysis: preterm SGA vs. full-term. Abbreviations: CI, confidence interval; DBP, diastolic blood pressure; SBP, systolic blood pressure; SCr, serum creatinine; SD, standard deviation; SGA, small for gestational age; Std, standardized.

We compared the SBP and DBP in preterm SGA and full-term groups. A total of four studies measured and compared the blood pressure [24,33,38,40]. The SBP was significantly higher in the 94 preterm-born patients compared to the 1739 full-term-born counterparts (SMD 0.41, 95% CI 0.12 to 0.70) (Figure 6). This trend was similar in the DBP (SMD 0.28, 95% CI 0.05 to 0.51).

There was no significant difference in the level of SCr between the preterm SGA and full-term survivors (SMD 0.18, 95% CI −0.24 to 0.59; 38 preterm-born and 75 full-term controls) (Figure 6).

#### 3.5.2. AGA

**Figure 7:**
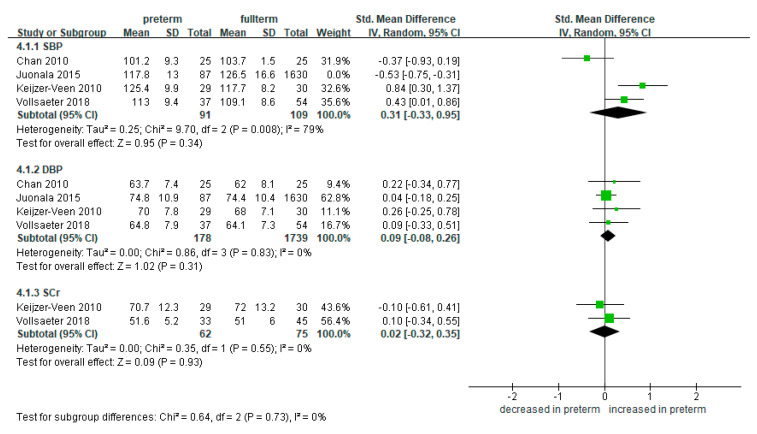
Forest plot of subgroup meta-analysis: preterm AGA vs. full-term. Some studies with high disparity were removed from analysis to control high heterogeneity. Abbreviations: AGA, appropriate for gestational age; CI, confidence interval; DBP, diastolic blood pressure; SBP, systolic blood pressure; SCr, serum creatinine; SD, standard deviation; Std, standardized.

We compared blood pressure and SCr in preterm AGA and full-term groups. A total of four studies measured and compared the blood pressure [24,33,38,40]. The SBP was not significantly higher in the 178 preterm-born patients compared to the 1739 full-term-born counterparts (SMD 0.31, 95% CI −0.33 to 0.95) (Figure 7). This trend was similar in the DBP (SMD 0.09, 95% CI −0.08 to 0.26).

There was no significant difference in the level of SCr between the preterm AGA and full-term survivors (SMD 0.16, 95% CI −0.03 to 0.35; 62 preterm-born and 75 full-term controls) (Figure 7).

The SBP, DBP, and SCr were not significantly different in the preterm-born patients compared to the full-term-born counterparts (Figure 7).

## 4. Discussion

In the present meta-analysis, in comparison to their full-term born counterparts, preterm survivors showed significantly decreased GFR, increased albuminuria, decreased renal mass and adjusted volume, decreased ERPF, and higher SBP and DBP. However, the previously known and most widely used serum biomarkers of renal function (i.e., BUN, SCr, and cystatin C) did not significantly differ between the two groups. Similarly, although the SBP and DBP were consistently more elevated in the preterm-born group, the serum levels of renin, a biomarker of renal hypertension, did not significantly differ.

SCr and BUN levels may not reflect mild renal impairment as long as the renal function is maintained by the remnant nephrons. It is well known that the SCr concentration increases only at a reduction of about 50% in the GFR [50]. Moreover, the level of these markers could be affected by not only renal function but also body muscle mass, protein intake, endogenous protein catabolism, and state of hydration [51]. Regarding SCr, five out of six studies involved in the analysis reported no difference [8,24,25,28,38,44]. As these studies had varying follow-up periods, the results might have been affected by the muscle mass in younger patients. Cystatin C is a biomarker more independent of age or muscle mass compared to SCr, and it has been suggested that it might predict the risk of developing CKD at a mild, preclinical state of renal dysfunction [52,53]. There were three studies that investigated this biomarker in the long term [24,25,28], and one study reported a significant elevation in the preterm-born group [28]. Since the number of studies involved is very small, the clinical usefulness of cystatin C in this population remains to be seen. Calculated GFR and ERPF were decreased in the preterm-born group, although these comparisons showed a rather borderline significance. A decrease in these factors can be explained by the reduced renal mass which will be discussed later. Urinary levels of microalbumin were measured in four studies, all of which reported an increase [8,25,36,44]. This implicates that patients may be developing microalbuminuria even though it may not be detectable by simple urine dipstick tests.

Kidney lengths and volumes are often used as surrogate markers of nephron mass [54]. All three studies that included comparisons of kidney volumes and lengths uniformly reported a significant decrease in the absolute and relative renal volumes [25,28,42], and this was also supported by shortened kidney lengths in two studies [28,42]. As we understand, the mechanism of decrease in renal mass can be attributed to renal insult due to nephrotoxic drugs, poor circulation accompanied by situations such as sepsis, heart dysfunction, and respiratory impairment in preterm infants [55,56,57,58]. Moreover, further pathophysiology of decreased renal mass in preterm-born patients was recently suggested in a case series by Kim et al. [10]. The authors reported radiologic evidence of cystic dysplasia of the kidneys in a series of patients born extremely preterm and postulated that such a change can be interpreted as another form of unique developmental dysplasia in prematurity, such as bronchopulmonary dysplasia and periventricular leukomalacia [10]. It is therefore assumable that nephronal loss from either or both episodes of renal insult or unique histologic dysplasia may reduce the reservoir of the kidney function, which in turn would make the preterm-born survivors more vulnerable to insults, such as volume depletion, trauma, overweight, and hypertension. The decrease in renal mass, however, may not be detectable with serum levels of common biomarkers and thus required a regular imaging, most commonly by ultrasonography.

In this meta-analysis, blood pressure was investigated by several studies [8,24,25,26,27,29,30,31,32,33,37,38,39,40,41,43,44,46,47,48], and the results were uniform in that the preterm-born group had higher SBP and DBP both in single-time and ambulatory monitored measures. These results were consistent with the previous meta-analyses [59,60,61,62]. Although SGA and AGA were not distinguished for their difference in most of the previous studies, our study showed that higher blood pressure associated with preterm survivors was observed only in the SGA and not in the AGA group. We postulate that age-appropriate development may have more impact than the birth age per se. Further studies with a larger number of patients with comparison analysis on SGA vs. AGA groups are required for supporting this assumption. There are possible mechanisms that may explain the association between preterm birth, SGA, and high blood pressure. It has been understood that premature birth may induce changes in vascular resistance and endothelial function [33,63]. In addition, both preterm birth and intrauterine growth restriction are related to reduced number of nephrons [64,65]. Kidneys with fewer nephrons may lead to a diminished filtration surface area, resulting in limitation of sodium excretion, causing raised blood pressure and reduction in renal adaptive capacity [12,65]. Moreover, compensatory mechanisms including glomerular hypertrophy and mesangial proliferation could lead to hyperfiltration [9]. As a body of literature supports, hypertension is a strong risk factor for developing CKD, and effective blood pressure control has been shown to delay disease progression [16,66].

The results in this study must be interpreted with caution due to the following limitations. First, the methods of measuring the markers were not uniformly controlled and instead may significantly differ by each center. Second, some studies were excluded due to a lack of accessible raw data, and there remains the possibility of existing case reports or series that were not accessible. Third, further subgroup comparisons according to gender, age, and birth weight were not available due to the limited number of eligible studies. Fourth, there were studies which investigated different factors from the same cohort [38,42,45,47]. Although each study was focused on different factors, it may have involved some duplication of patient data. Lastly, due to the limited number of studies, some comparisons had to contain only two or three studies per item. Sometimes, the studies were included in spite of high heterogeneity. Lacking randomized controlled trials by nature (since prematurity itself cannot be randomized) may have also contributed to the high disparity. Although we used a random effects model to compromise as we could, the high heterogeneity and small number of studies may decrease the validity of the analyses. Nevertheless, considering the extreme paucity of long-term cohorts in this topic, we considered it valuable to demonstrate as many relevant studies as available. Further meta-analyses containing studies with more patients would be powerful in verifying the results of the present study.

Nevertheless, the present meta-analysis implied that premature birth may negatively impact the renal function in the long term, presumably due to decreased nephronal mass caused by insults in the period of nephrogenesis. Since the patients are still of relatively younger ages, earlier signs of mild renal impairment might not be detectable through commonly used laboratory biomarkers. Instead, radiological monitoring of the renal length and volume may be more helpful in predicting chronic renal impairment.

## 5. Conclusions

In this meta-analysis of long-term cohorts, the preterm-born patients, compared to the full-term-born controls, had decreased renal mass, decreased ERPF, increased microalbuminuria, and higher blood pressure. However, traditional biomarkers, such as serum levels of creatinine, BUN, cystatin C, and renin, were not significantly different between preterm-born patients and full-term-born controls. For blood pressure, as compared with full-term controls, patients who were born as AGA had comparable outcomes, whereas SGA patients had significantly increased blood pressure. Whether AGA patients are at similar risks to full-term controls in other biomarkers or radiologic aspects requires further validation with more studies.

We hope this study could arouse awareness of the notion that the preterm-born population with apparently normal renal function may be subject to a decreasing renal mass. Serial sonographic measurement of the kidneys and continuous follow-up over a long-term period with strict blood pressure control may help the patients cope with their decreased nephronal reservoir and protect them from long-term risks of CKD.

## Figures and Tables

**Table 1:** Characteristics of all studies included in the meta-analysis.

Author Year	Study Groups	*n*	GA (Weeks) ^1^	Birth Wt (g) ^1^	SGA *n* (%)	BMI (kg/m^2^) ^1^	Age at FU (Years) ^1^
South, 2019 [8]	Preterm	96	27.8 ± 2.6	1048 ± 276	9 (9.4)	22.1 ± 5.1	14
	Full-term	43	39.7 ± 1.1	3458 ± 451	3 (7.0)	21.5 ± 3.5	14
Vollsaeter, 2018 [24]	Preterm (SGA)	20	28.0 ± 1.6	724 ± 143.2	20 (100.0)	17.6 ± 3.1	11.3 ± 0.9
	Preterm (AGA)	37	26.1 ± 1.2	918 ± 151.5	0 (0.0)	17.5 ± 2.2	11.4 ± 1.0
	Full-term (AGA)	54	n/a	3701 ± 434.1	0 (0.0)	17.8 ± 2.7	11.7 ± 1.5
Paquette, 2018 [25]	Preterm	92	27.1 ± 1.3	955 ± 223	6 (6.5)	22.6 ± 3.8	23.2 ± 2.2
	Full-term	92	39.5 ± 1.1	3401 ± 376	6 (6.5)	23.7 ± 4.4	23.2 ± 2.3
Kowalski, 2018 [26]	Preterm	76	27 ± 1	904 ± 161	12 (15.8)	23.0 ± 4.7	18.2 ± 1.3
	Full-term (AGA)	42	39 ± 1	3435 ± 470	0 (0.0)	23.2 ± 3.7	18.6 ± 0.9
Bonamy, 2017 [27]	Preterm	171	25.4 ± 1.0	786 ± 169	22 (12.9)	14.7 ± 1.7	6.6 ± 0.2
	Full-term	172	39.8 ± 1.2	3595 ± 465	3 (1.7)	16.0 ± 2.1	6.7 ± 0.2
Starzec, 2016 [28]	Preterm	64	27 ± 5.8	875 ± 406	19 (29.7)	n/a	11 ± 1.0
	Full-term	36	40 ± 1.5	3570 ± 717	2 (5.6)	n/a	10.7 ± 1.3
Gilarska, 2016 [29]	Preterm	67	27 ± 2.3	850 ± 128	n/a	n/a	11.0 ± 0.3
	Full-term	38	39.8 ± 1.4	3571 ± 538	n/a	n/a	10.6 ± 0.9
Washburn, 2015 [30]	Preterm	124	27.8 ± 2.6	1056 ± 272	n/a	22.8 ± 5.2	14
	Full-term	44	39.6 ± 1.1	3457 ± 446	n/a	22.8 ± 5.2	14
Mathai, 2015 [31]	Preterm	22	n/a	n/a	n/a	28.6 ± 4.3	35.8 ± 1.2
	Full-term	14	n/a	n/a	n/a	26.2 ± 4.4	35.6 ± 1.1
Lewandowski, 2015 [32]	Preterm	30	30.5 ± 2.7	1295.6 ± 304.5	n/a	26.3 ± 7.2	26.6 ± 1.0
	Full-term	60	39.6 ± 0.8	3411.2 ± 319.0	n/a	23.0 ± 3.3	26.2 ± 1.9
Juonala, 2015 [33]	Preterm (SGA)	39	n/a	n/a	39 (100.0)	27.3 ± 5.1	40.7 ± 4.3
	Preterm (AGA)	87	n/a	n/a	0 (0.0)	27.3 ± 5.6	41.3 ± 4.9
	Full-term	1630	n/a	n/a	n/a	26.5 ± 5.0	41.3 ± 4.9
Gunay, 2014 [34]	Preterm	65	35.7 ± 0.4	2521.2 ± 119.2	n/a	n/a	9.0 ± 3.2
	Full-term	65	38.5 ± 0.6	3328.9 ± 97.8	n/a	n/a	9.5 ± 2.7
Bassareo, 2013 [35]	Preterm	12	26.8 ± 2.0	927.3 ± 67.5	n/a	21.6 ± 6	23.9 ± 3.2
	Full-term	12	39.8 ± 0.3	3256.7 ± 151.5	n/a	21.5 ± 7	23.8 ± 2.9
Kwinta, 2011 [36]	Preterm	78	27.3 ± 2.2	866.7 ± 140.7	22 (28.2)	n/a	6.7 ± 0.4
	Full-term	38	40.0 ± 1.5	3591.3 ± 304.4	2 (5.3)	n/a	6.8 ± 0.7
Lazdam, 2010 [37]	Preterm	71	30.3 ± 2.5	1303.4 ± 278.8	n/a	24.4 ± 4.3	24
	Full-term	38	n/a	n/a	n/a	23.1 ± 2.6	24
Keijzer-Veen, 2010 [38]	Preterm (SGA)	21	30.6 ± 1.1	858 ± 132	21 (100.0)	21.7 ± 2.6	20.7 ± 0.3
	Preterm (AGA)	29	29.5 ± 1.4	1489 ± 257	0 (0.0)	22.1 ± 2.8	20.7 ± 0.4
	Full-term (AGA)	30	40.2 ± 1.3	3632 ± 489	0 (0.0)	22.9 ± 2.8	20.7 ± 0.8
Hovi, 2010 [39]	Preterm	118	29.2 ± 2.2	1138 ± 224	39 (33.1)	22.0 ± 3.8	18–27
	Full-term (AGA)	120	40.1 ± 1.0	3623 ± 479	0 (0.0)	23.2 ± 3.6	18–27
Chan, 2010 [40]	Preterm (SGA)	14	30.3 ± 1.6	929 ± 200	14 (100.0)	18.0 ± 4.2	13.3 ± 1.1
	Preterm (AGA)	25	29.5 ± 2.6	1492 ± 636	0 (0.0)	19.7 ± 2.4	14.3 ± 1.0
	Full-term (AGA)	25	39.8 ± 1.9	3366 ± 433	0 (0.0)	18.7 ± 2.3	13.6 ± 1.7
Evensen, 2009 [41]	Preterm (SGA)	14	32 (27–35) ^2^	1415 (800–1500) ^2^	14 (100.0)	23.9 ± 3.4	18.4 ± 0.7
	Preterm (AGA)	23	28 (24–31) ^2^	1210 (820–1490) ^2^	0 (0.0)	21.2 ± 3.4	18.1 ± 0.5
	Full-term (AGA)	63	40 (37–42) ^2^	3700 (2670–5140) ^2^	0 (0.0)	23.2 ± 3.2	18.6 ± 0.8
Keijzer-Veen, 2007 [42]	Preterm (SGA)	23	30.6 ± 1.0	859 ± 126	23 (100.0)	21.6 ± 2.5	20.7 ± 0.3
	Preterm (AGA)	29	29.5 ± 1.4	1489 ± 257	0 (0.0)	22.1 ± 2.8	20.7 ± 0.4
	Full-term (AGA)	30	40.2 ± 1.3	3632 ± 489	0 (0.0)	22.9 ± 2.8	20.7 ± 0.8
Bonamy, 2007 [43]	Preterm	39	28.9 ± 1.6	1106 ± 305	20 (51.3)	16.8 ± 2.8	9.1 ± 1.7
	Full-term	21	40.3 ± 1.0	3704 ± 404	0 (0.0)	16.2 ± 2.0	9.7 ± 1.5
Rodríguez-Soriano, 2005 [44]	Preterm	40	27.6 (23–35) ^3^	845 (540–1000) ^3^	13 (32.5)	16.0 ± 2.3	8.6 ± 1.8
	Full-term	43	n/a	n/a	n/a	19.3 ± 2.7	8.5 ± 1.8
Kistner, 2005 [45]	Preterm (AGA)	14	30 (28–32) ^2^	1250 (950–2040) ^2^	0 (0.0)	n/a	26 ± 2
	Full-term (AGA)	17	n/a	3720 (3120–4220) ^2^	0 (0.0)	n/a	26 ± 2
Doyle, 2003 [46]	Preterm	156	28.8 ± 2.0	1098 ± 235	n/a	n/a	18+
	Full-term	60	40.0 ± 1.1	3493 ± 494	n/a	n/a	18+
Kistner, 2000 [47]	Preterm	15	n/a	1293 ± 283	n/a	23.4 ± 2.9	26 ± 1.9
	Full-term (AGA)	17	n/a	3720 ± 313	0 (0.0)	23.9 ± 3.1	26 ± 1.9
Siewert-Delle, 1998 [48]	Preterm	44	n/a	3045 ± 646	n/a	25.6 ± 3.0	49
	Full-term	336	n/a	3559 ± 526	n/a	25.7 ± 3.5	49
Vanpée, 1992 [49]	Preterm	8	28.2 ± 1.5	n/a	n/a	n/a	8
	Full-term	12	n/a	n/a	n/a	n/a	(2.0–25.3) ^4^

^1^ mean ± SD; ^2^ median (range); ^3^ mean (range); ^4^ range. Abbreviations: AGA, appropriate for gestational age; BMI, body mass index; FU, follow-up; GA, gestational age; n/a, not available; SGA, small for gestational age; Wt, weight.

## Data Availability

The raw data supporting this meta-analysis were collected from previously published studies, which have been cited. The datasets generated and/or analyzed during the current study are available from the corresponding author on reasonable request.

## Supplementary Materials

The following are available online at https://www.mdpi.com/1660-4601/18/6/2951/s1, Table S1: Search strategy for PubMed and EMBASE, Table S2: PRISMA checklist, Table S3: The Newcastle–Ottawa scale (NOS) for case–control studies.
