# Supplementary material for: The Long-Term Effect of Preterm Birth on Renal Function: A Meta-Analysis
Source: Int J Environ Res Public Health. 2021 Mar 13;18(6):2951. doi: 10.3390/ijerph18062951 (PMC8001027; doi:10.3390/ijerph18062951)
Supplement: Supplementary file 1 [file ijerph-18-02951-s001.zip › ESM/ESM1.pdf]

Table S1. Search strategy for PubMed and EMBASE

PubMed

No.	Query	Results
#1	((((infant, premature[MeSH Terms]) OR prematur*[Text Word]) OR pre-matur*[Text Word]) OR preterm[Text Word]) OR pre-term[Text Word]	199910
#2	((low birth weight[MeSH Terms]) OR "low birth weight infant*"[Text Word]) OR LBW[Text Word]	35377
#3	#1 OR #2	217127
#4	(((((((kidney NEXT/2 (function*[Text Word] OR failure*[Text Word] OR disease*[Text Word] OR insufficien*)[Text Word])) OR (renal NEXT/2 (function*[Text Word] OR failure*[Text Word] OR disease*[Text Word] OR insufficien*)[Text Word])) OR "glomerular filtration rate*"[Text Word]) OR hypertension[Text Word]) OR proteinuria[Text Word]) OR microalbuminuria[Text Word]) OR nephron*[Text Word]	438902
#5	#3 AND #4	8913
#6	#5 AND Language [English]	8013

EMBASE

No.	Query	Results
#1	‘prematurity‘/exp OR ‘prematurity‘	111950
#2	prematur*:ab,ti,kw	184738
#3	‘,pre-matur*‘:ab,ti,kw	579
#4	preterm:ab,ti,kw	91586
#5	‘pre-term‘:ab,ti,kw	3881
#6	‘low birth weight‘:ab,ti,kw	32961
#7	‘LBW‘:ab,ti,kw	4723
#8	#1 OR #2 OR #3 OR #4 OR #5 OR #6 OR #7	291471
#9	kidney NEXT/2 (function* OR failure* OR disease* OR insufficien*):ab,ti,kw	135180
#10	renal NEXT/2 (function* OR failure* OR disease* OR insufficien*):ab,ti,kw	320594
#11	‘glomerular filtration rate‘:ab,ti,kw	55080
#12	hypertension:ab,ti,kw	555152
#13	proteinuria:ab,ti,kw	53665
#14	microalbuminuria:ab,ti,kw	13100

#15	nephron*:ab,ti,kw	19764
#16	#9 OR #10 OR #11 OR #12 OR #13 OR #14 OR #15	960957
#17	#8 AND #16	17938
#18	#17 AND ([english]/lim)	16375
