# Supplementary material for: The Long-Term Effect of Preterm Birth on Renal Function: A Meta-Analysis
Source: Int J Environ Res Public Health. 2021 Mar 13;18(6):2951. doi: 10.3390/ijerph18062951 (PMC8001027; doi:10.3390/ijerph18062951)
Supplement: Supplementary file 1 [file ijerph-18-02951-s001.zip › ESM/ESM3.pdf]

Table S3. The Newcastle-Ottawa Scale (NOS) for case-control studies [20]

Author, year	Selection			Comparability			Exposure		Total
	Case def[1]	Case rep[2]	Cont. sel[3]	Cont. def[4]	1-conf[5]	More[6]	Ascert[7]	Same mt[8]	NR Rate[9]	
South, 2019 [8]	1	1	0	1	1	0	1	1	0	6
Vollsaeter, 2018 [24]	1	1	0	1	1	1	1	1	1	8
Paquette, 2018 [25]	1	1	1	1	1	1	1	1	1	9
Kowalski, 2018 [26]	1	1	0	1	1	1	1	1	0	7
Bonamy, 2017 [27]	1	1	0	1	1	1	1	1	0	7
Starzec, 2016 [28]	1	1	0	1	1	0	1	1	0	6
Gilarska, 2016 [29]	1	1	0	1	1	0	1	1	0	6
Washburn, 2015 [30]	1	1	0	1	1	0	1	1	0	6
Mathai, 2015 [31]	1	1	0	1	1	1	1	1	0	7
Lewandowski, 2015 [32]	1	1	1	1	1	1	1	1	0	8
Juonala, 2015 [33]	1	1	0	1	1	0	1	1	0	6

Gunay, 2014 [34]	1	0	0	1	1	1	1	1	0	6
Bassareo, 2013 [35]	1	1	0	1	1	1	1	1	0	7
Kwinta, 2011 [36]	1	1	0	1	1	0	1	1	0	6
Lazdam, 2010 [37]	1	1	0	1	1	1	1	1	0	7
Keijzer-Veen, 2010 [38]	1	1	0	1	1	0	1	1	0	6
Hovi, 2010 [39]	1	1	0	1	1	0	1	1	0	6
Chan, 2010 [40]	1	1	1	1	1	0	1	1	0	7
Evensen, 2009 [41]	1	1	0	1	1	1	1	1	0	7
Keijzer-Veen, 2007 [42]	1	1	1	1	1	0	1	1	0	7
Bonamy, 2007 [43]	1	1	0	1	1	0	1	1	0	6
Rodríguez-Soriano, 2005 [44]	1	1	0	0	1	1	1	1	0	6
Kistner, 2005 [45]	1	1	0	1	1	0	1	1	0	6
Doyle, 2003 [46]	1	1	0	1	1	0	1	1	0	6

Kistner, 2000 [47]	1	1	0	1	1	0	1	1	0	6
Siewert-Delle, 1998 [48]	1	1	1	1	0	0	1	1	0	6
Vanpée, 1992 [49]	1	0	0	1	1	0	1	1	0	5

[1]Case definition adequate?; [2]Representativeness of the cases; [3]Selection of controls; [4]Definition of controls; [5]Study control for most important confounder factor; [6]Study controls for additional factors; [7]Ascertainment of exposure; [8]Same method of ascertainment for cases and controls; [9]Non response rate.
